# Improving palliative care in selected settings in England using quality indicators: a realist evaluation

**DOI:** 10.1186/s12904-016-0144-1

**Published:** 2016-08-02

**Authors:** Steve Iliffe, Nathan Davies, Jill Manthorpe, Peter Crome, Sam H Ahmedzai, Myrra Vernooij-Dassen, Yvonne Engels

**Affiliations:** 1 Research Department of Primary Care & Population Health, University College London, Royal Free Campus, Rowland Hill Street, London, NW3 2PF UK; 2 Social Care Workforce Research Unit, King’s College London, Strand, London, WC2B 6NR UK; 3 Department of Oncology and Metabolism, School of Medicine and Biomedical Science, The University of Sheffield, Sheffield, S10 2RX UK; 4 Scientific Institute for Quality of Healthcare, Radboud University Medical Centre, 6500 HB Nijmegen, The Netherlands

**Keywords:** Quality improvement, Quality indicators, Palliative care, Cancer, Dementia, Care homes, Primary care, Hospices, Realist evaluation

## Abstract

**Background:**

There is a gap between readily available evidence of best practice and its use in everyday palliative care. The IMPACT study evaluated the potential of facilitated use of Quality Indicators as tools to improve palliative care in different settings in England.

**Methods:**

1) Modelling palliative care services and selecting a set of Quality Indicators to form the core of an intervention, 2) Case studies of intervention using the Quality Indicator set supported by an expert in service change in selected settings (general practice, community palliative care teams, care homes, hospital wards, in-patient hospices) with a before-and-after evaluation, and 3) realist evaluation of processes and outcomes across settings. Participants in each setting were supported to identify no more than three Quality Indicators to work on over an eight-month period in 2013/2014.

**Results:**

General practices could not be recruited to the study. Care homes were recruited but not retained. Hospital wards were recruited and retained, and using the Quality Indicator (QI) set achieved some of their desired changes. Hospices and community palliative care teams were able to use the QI set to achieve almost all their desired changes, and develop plans for quality improvements. Improvements included: increasing the utility of electronic medical records, writing a manual for end of life care, establishing working relationships with a hospice; standardising information transfer between settings, holding regular multi-disciplinary team meetings, exploration of family carers’ views and experiences; developing referral criteria, and improvement of information transfer at patient discharge to home or to hospital.

Realist evaluation suggested that: 1) uptake and use of QIs are determined by organisational orientation towards continuous improvement; 2) the perceived value of a QI package was not powerful enough for GPs and care homes to commit to or sustain involvement; 3) the QI set may have been to narrow in focus, or more specialist than generalist; and 4) the greater the settings’ ‘top-down’ engagement with this change project, the more problematic was its implementation.

**Conclusions:**

Whilst use of QIs may facilitate improvements in specialist palliative care services, different QI sets may be needed for generalist care settings.

**Electronic supplementary material:**

The online version of this article (doi:10.1186/s12904-016-0144-1) contains supplementary material, which is available to authorized users.

## Background

The science of clinical decision-making is well developed but the science of the delivery of care is relatively new and unfamiliar to most health professionals [1]. As a consequence there are gaps between available evidence of best practice and its application in everyday care, across all disciplines within health care [2]. This includes palliative care [3, 4], which aims to “improve the quality of life of patients and families who face life-threatening illness, by providing pain and symptom relief, spiritual and psychosocial support from diagnosis to the end of life and bereavement” [5].

The ageing of the population and the longer survival of people with life-threatening conditions have resulted in rising demand for palliative care from patients with multiple and complex problems [6, 7]. For example, in Europe within the next decade the incidence and prevalence of cancers will increase by about 20 % whilst the prevalence of dementia is expected to double before 2050 [8, 9]. Meeting the palliative care needs of this growing population is a big task, since the aim is to optimise the quality of life of people who have complex, incurable and life-threatening health problems by addressing their physical, emotional, psychosocial and spiritual needs.

Palliative care for people with cancer is relatively well developed, in terms of its conceptual framework and evidence base [10]. The evidence base to guide practice with those dying with complex non-cancer conditions (for example, dementia) is less well developed, although now evolving [11]. The organisation of palliative care has been described extensively [12–14], but it is not clear how best to improve its quality, especially for non-cancer conditions.

Quality Indicators (QIs) can be used to assess where care can be improved. Campbell et al, [15], following Danabedian [16], describe QIs as evidence-based, explicitly defined and measurable items that evaluate and describe the structure, processes and outcomes of health care, and that can indicate problems in achievement of good quality care. They can be used to assess and feed back to professionals their actual performance against benchmarks, as a starting point for quality improvement [17]. QIs have been used effectively to assess and improve hospital care [18], primary care [19], and dementia care [20]. Several international studies have also developed QIs to improve the structures and process needed for the delivery of good quality palliative cancer and dementia care [21–23]. However, these indicator sets have not yet been widely applied in everyday clinical practice. This study developed and implemented a QI package to improve the organisation of palliative care in five European countries [24]. This paper describes the experiences of using a QI set to improve palliative care in different settings in one of the five countries, England.

## Methods

### Aim

The IMPACT project (IMplementation of Quality Indicators in PAlliative Care sTudy, 2011 to 2015) aimed to evaluate the potential of QIs as tools to improve palliative care for people with cancer or dementia in five European sites (England, Germany, Italy, Norway, and The Netherlands). We adopted Batalden and Davidoff’s definition of quality improvement [25] as “combined and unceasing efforts… to make changes that will lead to better patient outcomes (health), better system performance (care) and better professional development (learning)”.

### Study design

Evaluating the effect of QIs required research methods that were able to respond to the many factors that shape palliative care in general and its organisational processes in particular. Controlling these aspects in an international study with strict deadlines made a randomised controlled trial (RCT) impractical [26, 27]. RCTs of services are usually focused on one setting and often in one country, while this study explored and initiated QIs in several settings in multiple European countries with varying health care systems. A before-and-after (pre-test, post-test) design was used instead.

The stepwise development of the QI set for this study was informed by the UK Medical Research Council (MRC) framework for the development and evaluation of complex interventions to improve health [28] and guidance for applying research findings to end of life care [29]. Table 1 shows the intervention development tasks that were included in the IMPACT project; these included modelling the landscape of palliative care [30], identification of barriers to change [31], design of the evaluation study [32], achievement of expert consensus on QIs, [33], and clarification of commonalities and differences in palliative care by disease [34].

**Table 1:** Tasks of the IMPACT project

Year 1	Theory
	Modelling the organisation of palliative care in Europe [27]
Year 2	Modelling
	Identifying barriers and facilitators to successful interventions in focus groups and individual interviews 28]
	Identifying quality improvement strategies to improve the organisation of palliative care [29]
	Development of a QI set to assess the provision of palliative care [30]
Year 3	Pilot
	Pre-test: assessment of the organisation of palliative care, using QIs
	Implementation of strategies to improve the organisation of palliative care, tailored to national and setting-specific barriers and facilitators [31]
	Post-test: assessment of the organisation of palliative care, using QIs
Year 4	Evaluation and dissemination
	Process evaluation of the pilot
	Development of toolkit and manual about how to implement changes in the organisation of palliative care
	Other scientific output (papers, presentations, etc.)

### Ethical considerations

This study invited health and care professionals to participate in a quality improvement process, and those who participated were asked to give their written consent. Medical or care records were assessed by the health care professionals in the settings themselves. No patient data were recorded or used in a way that would allow the identity of patients to be recognised. The research team had an agreed protocol for responding to any potentially poor practice that they encountered.

### Theory and modelling

A qualitative description of palliative care models in Europe informed both the development of strategies to improve the organisation of palliative care using QIs. The QIs were derived from existing indicator sets and selected using a modified RAND Delphi procedure [35] involving a mix of clinicians and researchers active in palliative care [32].

The final set of 32 QIs used in the intervention study referred to: access to palliative care (specialist services, out of hours care, continuity of care), infrastructure of palliative care, assessment tools used by practitioners, personnel providing palliative care services (disciplines, teamwork, sharing information), documentation of clinical data, quality of care and its measurement, and education about palliative care.

Additional file 1 provides an overview of the quality indicators.

As an example of how these Qis were operationalised, the QIs on access to specialist palliative care services include the following questions:

- QI 1: Is a specialist palliative care team available 24 h/day, 7 days a week (24/7)?
- QI 2: Is specialist palliative care advice available 24/7?
- QI 3: Are bereaved relatives and/or professionals offered support during the bereavement process?

Full details of the development of the complete QI set are reported in van Riet Paap et al [33]. The present paper focuses on the application of the QI set in different settings in England, and the evaluation of the project.

The strategies to improve the organisation of palliative care were identified in an integrative review [36] that drew on both empirical and theoretical literature on implementation strategies [37]. Subsequently, interviews and focus group interviews with health and care professionals were used to adjust the strategies to national and setting-specific circumstances, in order to develop a toolkit of country- and setting-specific strategies to improve the organisation of palliative care.

### Intervention

We know that using feedback alone results in small to moderate effects on service performance [38]. In addition to individualised feedback, this study also used other strategies tailored to influence setting-specific barriers and facilitators, to promote change. These included educational interventions (didactic, experiential, mentoring), process mapping, feedback on performance using audit data, multi-disciplinary case discussions and multi-component approaches [33]. Many of these strategies have been tested before [1, 39], but not yet in the complex multidimensional field of palliative care. Quality Indicators and improvement strategies were developed for four types of settings (hospitals, care homes, hospices and community palliative care teams); no strategies were developed for general practice because of the failure to recruit interested practices. Settings which opted to participate did so on the understanding that the Quality Indicator set would be used as a formative rather than summative tool; that is, the purpose of using QIs was not to reach a judgement about the quality of the service provided, but to allow participants to identify, reflect on and work on weaknesses in their service, as they perceived them.

### Setting and participants

**Fig. 1:**
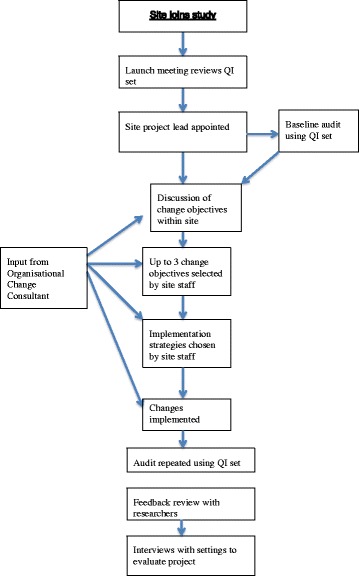
Flow chart of the pre-test, post-test study

The IMPACT team included members of the pan-European research group on detection and timely INTERvention in DEMentia (INTERDEM) and of the European Association of Palliative Care (EAPC), who used their research networks to purposefully select sites to take part in the intervention. Sites were excluded from participation in the study if they did not treat palliative patients aged 18 years or above, or if they had not provided any palliative care for the previous three years. Within these services, health and social care professionals were invited to participate in the pre-test –post-test study. Figure 1 shows the flow of the IMPACT pre-test, post-test study.

### Implementation

In the pre-test audit phase the QI set was discussed with professionals working in each setting to profile the palliative care services provided in each setting, and subsequently to identify aspects in their service that required improvement. At the launch meeting (see Fig. 1) a project lead for the site was selected. The project lead arranged the pre-test audit of the site’s palliative care services using the QI set. A web-based data registration system (Lime survey: www.limesurvey.org/) was used to capture answers about the use of QIs, allowing early comparison of data between settings and providing rapid feedback to participants. With feedback from the research team based on their responses, professionals in each site were then encouraged to set improvement objectives formulated in a SMART way (Specific, Measurable, Acceptable, Realistic and Timely) [40]. A consultant with expertise in service development helped each site to start and finish structured improvement projects [41]. In the intervention phase each site’s professionals then chose specific strategies, tailored to their situation, to improve the organisation of palliative care. After the intervention period (post-test phase), the palliative care service was re-assessed with the use of the same QI set.

### Evaluation and dissemination

A process evaluation was performed to study barriers and facilitators of the implementation process during the intervention period [42]. This process evaluation included an activity report which both the health and care professionals involved in the study and the consultant and researcher working with the site were asked to complete. These reports captured details of what was going to plan, what was not, and any barriers and facilitators facing the teams. To derive a deeper understanding of the barriers mentioned in these activity reports, semi-structured interviews were conducted among the professionals who participated in the implementation process at the end of the implementation period. In addition, field notes were kept by all researchers and consultants after each encounter at the implementation site, whether face-to-face, by telephone or by email.

To get a better understanding of the changes in the organisation of palliative care and the experiences of the health care professionals involved in the intervention, the qualitative data were analysed thematically [43]. A realist evaluation approach was used to synthesise the data from interviews, pre and post audits and field notes, in order to obtain a comprehensive picture of the contexts within which palliative care professionals worked, the mechanisms which promoted or impeded changes, and the consequent outcomes for each setting (without describing any outcome as primary or secondary, a priori). Realist evaluation works to the immediate priorities of empirical research, responds to the research brief, deals with the substantive issue and contributes to policy development, rather than aiming for methodological purity [44].

## Results

Table 2 shows recruitment and retention of sites in the English arm of the study. General practices could not be recruited to the study despite considerable effort over six months. A variety of approaches was used to recruit including the engagement of palliative care ‘champions’ and focusing upon trainee practitioners and practitioners recommended by palliative care teams and hospices for their interest in the topic.

**Table 2:** Recruitment and retention of settings in the English arm of the IMPACT study

	Setting				
Set up	General Practitioners (GPs)	Care Homes	Hospital wards	Community Palliative Care teams (PC)	Hospices
No. of invitations to participate	20 + (Palliative care champions, academic GPs, GP registrars)	Negotiations with care home chain (3 meetings) resulting in 3 Expressions of Interest	2 recruited through a specialist Dementias & neurodegenerative diseases research network (DeNDRoN)	2 recruited through a Clinical Research Network (CLRN)	2 recruited through CLRN
No. recruited	0	2	2	2	2
Baseline assessments	-	2	2	2	2
Baseline meetings	-	With managers not staff	With staff and team leader	With staff and management	With staff and management
Changes planned	-	• Workshops with staff on End of Life Care • Standardise use of pain scales	• Optimising IT use • Ring-fencing beds	See Table 3	See Table 3
Follow up	-		2	2	2
Content of follow up	-	Both Care Homes dropped out	Both sites completed follow up QI profile and interviews	Both sites completed follow up QI profile and interviews	Both sites completed follow up QI profile and part in interviews
Continued quality improvement…	-			1	1
	-		No plans	Continuing development	Continuing development

Care homes were recruited but not retained in the study. Three care homes were recruited from one commercial chain, with the support of their senior management, and two agreed to take part in the study. In one care home the manager spoke frankly about her difficulties in engaging the staff in palliative care and welcomed the opportunity to have staff workshops run by IMPACT consultants, but had moved to a different care home before these workshops could begin. In the second care home the manager explained at the launch meeting that it was the home’s policy to avoid providing palliative care to residents, preferring to transfer dying residents to other homes or to hospital. This manager agreed that wider staff use of pain scales would be an appropriate change for the care home to explore, but thereafter did not respond to further contacts with the research team.

Overall the participant sites performed relatively well against the Quality Indicator set. All had access to palliative care expertise outside normal office hours and at weekends, although not necessarily to all disciplines involved in multi-disciplinary palliative care teams. Regular meetings of multi-disciplinary teams occurred at all sites, as did training activities for team members. Medical records were not accessible across organisational boundaries, so those providing end of life care in a hospital setting would not be able to access records held in the nearby but organisationally separate hospice or community palliative care team. All sites provided bereavement support (albeit in different forms) to relatives, and most also offered bereavement support for staff. Opioids and co-analgesics could be obtained when needed. Communication about transfer of patients to other settings was systematised in all sites, using mixtures of written, electronic (email or fax) and verbal (telephone, face-to-face) methods. All sites had access to necessary equipment (drug administration pumps, anti-decubitus mattresses, stoma care supplies etc.) when needed. Single rooms for dying patients were available in all sites, except of course in community services supporting patients in their own homes. Use of standardised instruments (for example, for pain assessment, or assessment of emotional needs) varied considerably between sites. Similar variation was found in the systematic capture of relatives’ experiences of the services, and in use of palliative care guidelines.

Table 3 summarises changes planned in the implementation study in each site: ticks represent achievement of changes, as agreed by the research team and the participants at each site. Using the QI set hospital wards were recruited and retained, and achieved some of their desired changes. Hospices and community palliative care teams were able to achieve almost all their desired changes, and develop plans for future quality improvements. Changes that were not achieved were implementation of validated pain assessment scales and documenting conversations with patients. In all hospital ward, hospices and related community teams the changes pursued after applying the QI package took longer than the study’s six month follow-up period (by up to three months) because of intercurrent problems such as staff changes, funding difficulties and competing organisational demands, delaying data transfer on outcomes to the IMPACT research team.

**Table 3:** Changes planned in the implementation study in each site

Settings	Place	Changes planned and achieved✓
Care Home 1	Rural	More systematic use of pain scales
Care Home 2	Urban	Workshops with staff on End of Life Care
Hospital ward 1	Urban	Protect beds from other departments
		Improve utility of electronic records ✓
Hospital ward 2	Suburban	Writing an EOLC manual✓
		Establish links with hospice✓
		Establish rules for referral to other services (e.g. hospice) ✓
Community PC team 1	Urban/rural	Implement validated pain assessment scales
		Standardise transfer of information about discharge to GPs✓
		Hold regular Multi-disciplinary team meetings✓
Community PC team 2	Urban/rural	Documenting conversations with patients
		Explorations of family carer experiences✓
		Improve transfer of information to next setting✓
Hospice 1	Urban/rural	Implement validated pain assessment scores
		Standardise transfer of information on discharge and assessment by home care teams✓
		Develop criteria for referral of patients to other services (e.g. hospital) ✓
Hospice 2	Urban/rural	Documenting conversations with patients
		Explorations of family carer experiences✓
		Improve transfer of information on discharge to next setting✓

Table 4 shows the main headings of the realist evaluation, drawing together the research team’s conclusions on the impact of the contexts and the mechanisms on achievement of outcomes. Important contextual factors with a negative impact were the implementation of the controversial Health & Social Care Act 2012, which redefined the roles of general practitioners (in England) in particular, and the reorganisation of the care home chain involved in the study. The most evident mechanism with a negative impact on outcomes was ‘top down’ decision-making about joining the study; where senior management (in care homes or hospitals) decided to take part in the study, less senior staff resisted involvement.

**Table 4:** Realist evaluation: conclusions on the impact of the context and the mechanisms on achieving outcomes

Setting	Context	Mechanism	Outcome
General practice	Systemic organisational change in general practice (implementation of the Health & Social Care Act 2012)	GPs avoid additional tasks that increase work burden or are perceived outwith remit	No engagement with the project despite potential for general practice as a discipline and patient gain
Care homes	Top-down decision to involve care homes, during period of care home chain re-organisation	Care home managers avoid additional imposed tasks	No lasting engagement
Hospital wards	Top-down decision to involve hospital ward, and period of major staff turnover	Ward staff minimise involvement, momentum lost when key staff leave	Selection of indicators for change in order to minimise workload and maximise gain
Specialist palliative care schemes in hospices & communities	Competing demands in hospices & community teams, with funding challenges, periods of staff absence; but strong audit & research culture	Value of indicators appreciated, as supplement to existing audit & research culture	Changes made, effects seen, though extended implementation timescale required

## Discussion

The novel feature of the IMPACT project was the use of a QI set as part of a complex intervention to facilitate improvement in palliative care across a range of settings. The IMPACT QI set was comparable to the EUROPALL Quality Indicators [45] but more organisational and less clinical than those reviewed by Lorenz and colleagues [46].

The stepwise implementation used in this study enabled us to tailor the intervention at a setting and country level and so to translate the intervention into everyday clinical practice [12]. It followed the Batalden and Davidoff model of change, beginning with the generalisable scientific evidence that was applied in known contexts to give measureable performance improvements [22]. The realist evaluation focussed on developing explanatory models, using multiple data sources and methods, and sought to investigate contexts, mechanisms and outcomes in specific configurations [35].

Uptake and implementation of changes in organisation and practice identified using the QI set varied across settings, with maximal impact on in-patient hospices and community palliative care teams, and no impact in general practice or care homes. Although claims have been made that palliative care is the “territory” [47] of generalists the failure to engage general practices in the study is not surprising. The context (major recent changes in the British National Health Service across healthcare delviery and in general practice in particular, lack of funding for GPs to carry out palliative care) added to the known difficulties that GPs have in caring for dying patients [48], the challenge of maintaining palliative care skills [49], and the persistent taboo about discussing death [50].

Two objectives were not reached by any of the hospices and community paliative care teams: documentation of discussions with family carers; and routine use of validated pain assessment tools. Our impression is that the former had low priority compared with other communication tasks, such as transfering information if the patient moved settings. Pain management, of which formalised assessment tools are an important part, remains a problem in palliative care [51], and our participants’ inability to achieve their intended goal reflects this.

Realist evaluation suggested that four factors operated in the English sites:

1. uptake and use of QIs was determined by a history of organisational orientation towards continuous improvement, described by Flottorp and colleagues as “capacity for organisational change” [52];
2. the perceived value of the QI package offered was not powerful enough for GPs and care homes to commit to or sustain involvement, given their contexts;
3. the QI set may have been too specialist (in palliative care terms) in its focus and not useful enough for non-specialists (in care homes and in general practice) and;
4. the greater the settings’ ‘top-down’ engagement with this change project, the more problematic was its implementation.

These conclusions are consistent with Berwick’s view that any complex multi-component intervention is essentially a process of social change that is in turn influenced by leadership, changing environments, details of the implementation and organisational history [53]. It is also consistent with the argument that mechanisms are usually hidden and sensitive to variations in context [54].

The findings of this study should be treated cautiously. Althought case studies yield important lessons about service changes in complex organisational environments, and are therefore very appropriate to palliative care [55], their generalisability may by limited by their close attention to contexts. Before-and-after studies tend to over-estimate effect sizes [56], although this probably applies more to quantitative outcomes than to organisational processes, and realist evaluation has methodological problems that may limit its potential [57]. More involvement of generalists in the development of the QI set, with public and patient involvement too, might have resulted in a QI set that was more applicable to general practice and care homes.

## Conclusions

Nevertheless, the findings of this study can contribute to the further evolution of palliative care, as well as indicating where further research and development efforts are needed. For example, findings from the IMPACT project informed the European Declaration on Palliative Care in 2014 (www.palliativecare2020.eu/declaration/). In our view a single, generic QI set is unlikely to serve the needs of different settings, and further work is needed to find levers for change in general practice and care homes. Even in hospices and community palliative care teams the implementation of a QI set seemed to depend on the pre-existing research and development culture of the organisation. A range of methods for engaging services with less developed cultures is needed [58], especially if palliative care expertise is to be made available in England to those with non-cancer conditions, as will be recommended by the forthcoming NICE guidance (http://www.nice.org.uk/guidance/indevelopment/gid-cgwave0694)

## Abbreviations

EUROPALL, European Palliative care study; IMPACT, IMplementation of Quality Indicators in PAlliative Care sTudy; INTERDEM, interventions in dementia research group; MRC, Medical Research Council; NRES, National Research Ethics Service; QI, quality indicator; RCT, Randomised controlled trial; SMART, specific, measurable, acceptable, realistic and timely.

## Additional file

Additional file 1: Overview of IMPACT Quality Indicators. (DOCX 18 kb)
