# Supplementary material for: Improving palliative care in selected settings in England using quality indicators: a realist evaluation
Source: BMC Palliat Care. 2016 Aug 2;15:69. doi: 10.1186/s12904-016-0144-1 (PMC4970274; doi:10.1186/s12904-016-0144-1)
Supplement: Additional file 1: — Overview of IMPACT Quality Indicators. (DOCX 18 kb) [file 12904_2016_144_MOESM1_ESM.docx]

**Supplementary file 1 Overview of IMPACT Quality Indicators**

| ***1. Access to palliative care*** | |
| --- | --- |
| ***1a. Access and availability*** | |
| 1. | A **specialist palliative care team*** is available 24/7. |
| 2. | **Specialist palliative care*** advice is available 24/7 to professionals delivering palliative care. |
| 3. | Bereaved relatives and/or professionals involved in care of a person in need of palliative care are offered support during the bereavement process if they need or wish to have support. |
| ***1b. Out of hours care*** | |
| 4. | Opioids are accessible and available for persons in need of palliative care 24/7. |
| 5. | **Co-analgesics*** for symptom control are available to treat persons in need of palliative care 24/7. |
| ***1c. Continuity of care*** | |
| 6. | An (electronic) file of a person in need of palliative care is accessible to professionals in charge of the person 24/7. |
| 7. | At each transition between care settings, comprehensive information (including care goals and care plan) of a person in need of palliative care is be transferred to the professional(s) in charge in the next setting. |
| 8. | The professional in charge of the person is informed before a person in need of palliative care is discharged home or sent to the next setting. |
| 9. | Persons in need of palliative care have an assigned contact person who maintains regular contact with the person and their families, and ensures coordinated delivery of health and social care. |
| ***2. Infrastructure*** | |
| 10. | Specialised equipment (e.g. anti decubitus mattresses, suction equipment, stoma care, oxygen delivery, drug administration pumps, hospital beds, etc.) is available to persons in need of palliative care. |
| 11. | Single bedrooms are available for persons who are dying and who wish to have one. |
| 12. | Family members and friends are able to visit the dying person without restrictions of visiting hours. |
| 13. | There are facilities for relatives to stay overnight with their dying relative. |
| 14. | There is a private area for saying goodbye to the deceased, nearby or on the ward/unit where the person died. |
| ***3. Assessment tools*** | |
| 15. | There is a regular assessment of pain and other symptoms **using a validated instrument**. |
| ***4. Personnel*** | |
| ***4a. Team*** | |
| 16. | The multidisciplinary **team*** that delivers palliative care services consists of at least: |
|  | a) a physician and nurse; |
|  | b) and has access to one or more of the following professionals: physiotherapist, psychologist, occupational therapist, social worker, chaplain, dietician. |
| 17. | There is a weekly multidisciplinary meeting with at least the physician and nurse in charge of the person in need of palliative care to review treatment and care plans. |
| ***4b. Sharing information*** | |
| 18. | The file of the person in need of palliative care contains documentation of a discussion with the person or representative (if the person lacks capacity e.g. is unable to communicate) about: |
|  | a) medical condition; |
|  | b) goals for treatment; |
|  | c) the **physical***, psychosocial and spiritual needs of the person and family caregiver; |
|  | d) an advance directive or advanced care plan; |
|  | e) **end-of-life decisions***; |
|  | f) the intention to return home or to another facility from the place where the person is currently staying. |
| ***5. Documentation of clinical data*** | |
| ***5a. Clinical records*** | |
| 19. | The file of the person in need of palliative care contains a medication list that is accessible to the professionals caring for the person. |
| ***5b. Timely documentation*** | |
| 20. | Within 48 hours of admission to the service, the file of the person in need of palliative care contains documentation of the initial assessment of: |
|  | a) pain and other symptoms, using **a validated instrument***; |
|  | b) psychosocial and spiritual needs; |
|  | c) persons preferences, wishes and needs; |
|  | d) capacity to be involved in the decision making process. |
| ***6. Quality*** | |
| 21. | Family and caregiver experiences of the palliative care service are assessed/evaluated/recorded. |
| 22. | An end-of-life care pathway (such as the Liverpool Care Pathway) was used for the last 3 days of life of a person in need of palliative care. |
| ***7. Education*** | |
| 23. | All professionals that deliver palliative care services receive accredited training in palliative care, appropriate to their discipline. |
